# Evaluating the inhibition of IL-17A and TNFα in a cartilage explant model cultured with Th17-derived cytokines

**DOI:** 10.1016/j.jtauto.2024.100231

**Published:** 2024-01-07

**Authors:** Solveig Skovlund Groen, Anne-Christine Bay-Jensen, Christian S. Thudium, Morten H. Dziegiel, Marie Skougaard, Simon Francis Thomsen, Signe Holm Nielsen

**Affiliations:** a Immunoscience, Nordic Bioscience, Herlev, Denmark; b Department of Biomedical Sciences, University of Copenhagen, Copenhagen, Denmark; c Department of Clinical Immunology, Rigshospitalet, Copenhagen University Hospital, Copenhagen, Denmark; d The Copenhagen Center for Translational Research, Bispebjerg and Frederiksberg Hospital, Copenhagen, Denmark; e The Parker Institute, Bispebjerg and Frederiksberg Hospital, Frederiksberg, Denmark; f Department of Clinical Immunology, Aarhus University Hospital, Aarhus, Denmark; g Department of Dermatology, Bispebjerg Hospital, Copenhagen, Denmark; h Department of Biotechnology and Biomedicine, Technical University of Denmark, Kgs. Lyngby, Denmark

**Keywords:** Th17 differentiation, Interleukin-17a, Extracellular matrix, Biomarker, Tissue remodeling, Th17 cells

## Abstract

**Introduction:**

T-helper 17 (Th17) cells produce IL-17A playing a critical role in activating the pathogenic chain leading to joint tissue inflammation and destruction. Elevated levels of Th17 cells and IL-17A have been detected in skin lesions, blood, and synovial fluid from patients with psoriatic arthritis (PsA) and ankylosing spondylitis (AS). Moreover, IL-17A inhibitors suppress disease activity in psoriasis, PsA and AS, supporting the evidence of IL-17A contributing to the disease pathogenesis. Although, IL-17A inhibitors are widely approved, it remains unclear how the inhibitory effect of IL-17A alters the extracellular matrix (ECM) of the joint in a Th17-conditioned inflammatory milieu. Therefore, the aim of this study was to establish a cartilage model cultured with conditioned medium from Th17 cells and inhibitors to explore the effect of IL-17A inhibition on joint tissue remodeling.

**Methods:**

Naïve CD4^+^ T cells from healthy human buffy coat were differentiated into Th17 cells, followed by Th17 cell activation to secrete Th17-related cytokines and molecules into media. The activated Th17 cells were isolated from the conditioned media (CM) and analyzed using flow cytometry to verify Th17 cell differentiation. The CM were assessed with ELISA to quantify the concentrations of cytokines secreted into the media by the Th17 cells. Healthy bovine cartilage explants were cultured with the Th17-CM and treated with IL-17A and TNFα inhibitors for 21 days. In harvested supernatant from the cartilage cultures, MMP- and ADAMTS-mediated biomarker fragments of type II collagen, aggrecan, and fibronectin were measured by ELISA to investigate the ECM remodeling within the cartilage tissue.

**Results:**

Th17-CM stimulated a catabolic response in the cartilage. Markers of type II collagen and aggrecan degradation were upregulated, while anabolic marker of type II collagen formation remained on similar levels as the untreated explants. The addition of IL-17A inhibitor to Th17-CM decreased the elevated type II collagen and aggrecan degradation, however, degenerative levels were still elevated compared to untreated group. The addition of TNFα inhibitor completely reduced both type II collagen and aggrecan degradation compared to untreated explants. Moreover, the TNFα inhibitor treatment did not alter the type II collagen formation compared to untreated group.

**Conclusion:**

This study suggests that inhibition of IL-17A in Th17-conditioned cartilage tissue only partially reduced the MMP-mediated type II collagen degradation and ADAMTS-mediated aggrecan degradation, while the TNFα inhibitor treatment fully reduced both MMP- and ADAMTS-mediated ECM degradation. This exploratory study where ECM biomarkers are combined with Th17-conditioned *ex vivo* model may hold great potential as output for describing joint disease mechanisms and predicting structural effects of treatment on joint tissue.

## 1 Introduction

T-helper 17 cells (Th17, Interleukin(IL)-17-secreting CD4^+^ T cells) play a central role in both tissue homeostasis and inflammation during clearance of extracellular bacteria and fungi [1]. They are the best-known producers of IL-17A and IL-17F, where IL-17A is the most well-studied cytokine and has been linked to the pathogenesis of several inflammatory diseases, such as psoriasis, psoriatic arthritis (PsA), and ankylosing spondylitis (AS) [2]. In peripheral blood from psoriasis and PsA patients, levels of IL-17+ CD4^+^ T cells have shown to be higher compared to healthy donors. Similarly, levels of IL-17A were elevated in both PsA and psoriasis patients compared to healthy [3]. Moreover, the blockade of IL-17A with monoclonal antibodies (anti-IL-17A) in psoriasis, PsA, and AS has proven to be a highly effective therapeutic approach [[4], [5], [6], [7], [8], [9], [10], [11]].

The Th17-derived effector cytokines are mainly known to be IL-17A, IL-17F, IL-22, and tumor necrosis factor alpha (TNFα). Their effects on the joint tissue are diverse through orchestrating various phases of inflammatory disease processes, which can lead to structural erosions and ankylosis of the joints [12]. Numerous inflammatory mediator and effector molecules are secreted in response to the Th17-derived cytokines, including matrix-degrading enzymes, A Disintegrin and Metalloproteinase with Thrombospondin motifs (ADAMTSs) and Matrix Metalloproteinases (MMPs), which are responsible for degrading components of the extracellular matrix (ECM), such as collagens and proteoglycans. Increased production of MMPs by IL-17 leads to degradation of the ECM, contributing to tissue destruction of cartilage, bone, and synovial tissue, consequently damaging the joint [13]. In a study by Margheri et al. (2021), conditioned media (CM) from activated Th17 cells upregulated the expression and activity of MMP-2 and -9, which stimulated cartilage invasion and degradation by synovial fibroblasts. Furthermore, they showed that adding secukinumab (anti-IL-17A) prevented cartilage degradation induced by Th17-CM [14]. Several studies have demonstrated that IL-17A stimulates MMP-secretion activating joint tissue remodeling and destruction including cartilage degradation [[15], [16], [17]]. Likewise, IL-17F has proven to activate degradation of the ECM in cartilage by enhancing the expression of MMPs and decreasing the production of type II collagen and aggrecan [18].

Despite the advancements made in understanding the role of IL-17A and other Th17-derived cytokines in joint tissue pathology, several knowledge gaps still exist. Particularly, our comprehension of the precise impact of Th17-derived cytokines on the remodeling of joint tissue, including their effects on cartilage metabolism. The intricate interplay between IL-17A and other cytokines, such as TNFα, and their respective contributions to joint damage necessitate further investigation. Similarly, the precise effects of anti-IL-17A and anti-TNFα treatment on inhibiting protease activity altering the ECM remodeling within the joint tissue remain unknown.

As IL-17A is a registered therapeutic target in psoriasis, PsA, and AS, gaining novel knowledge on how Th17-derived cytokines drive joint damage could not only advance our understanding of disease mechanisms, but also help identifying potential therapeutic targets which can be used in combination with existing therapies to improve patient outcomes [19].

Consequently, this study aimed to investigate how IL-17A inhibition affects ECM remodeling of the joint in a Th17-conditioned inflammatory milieu. To explore this, healthy bovine cartilage explants were cultured with Th17-CM from activated Th17 cells and treated with IL-17A and TNFα inhibitors for 21 days. In the harvested supernatant from cartilage cultures, protease-mediated biomarker fragments of type II collagen, fibronectin, and aggrecan were measured by enzyme-linked immunosorbent assays (ELISAs) to investigate the ECM remodeling in cartilage tissue.

## 2 Material and methods

**Fig. 1:**
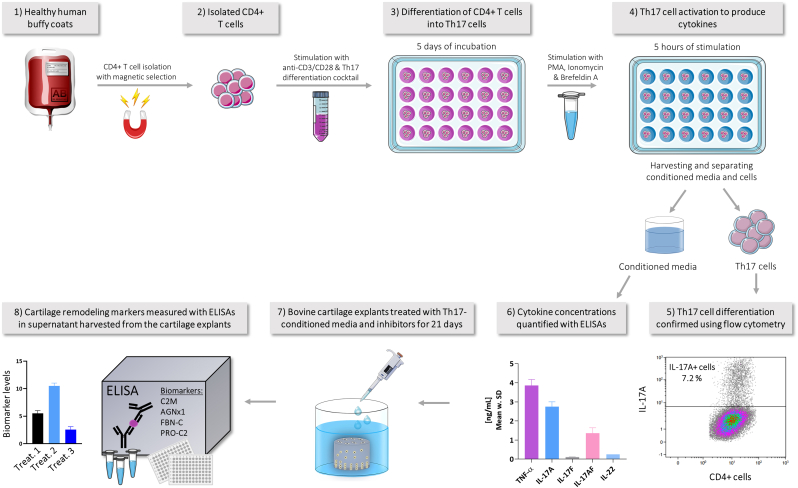
**Overall experimental procedure**. CD4^+^ T cells were isolated from healthy human buffy coats with magnetic negative selection and treated for 5 days with anti-CD3/CD28 including Th17 cell differentiation cocktail. After 5 days, Th17 cells were activated with PMA, ionomycin and brefeldin A for 5 h to produce Th17-related cytokines including IL-17. Then, Th7 cells were separated from the conditioned media (CM) and analyzed using flow cytometry to verify Th17 cell differentiation. The CM were assessed with ELISAs to quantify the concentrations of cytokines secreted into the media by the Th17 cells. Cartilage explants were cultured with the Th17-CM including inhibitors for 21 days. Finally, supernatant from the cartilage explants was analyzed by ELISAs to assess biomarkers levels reflecting ECM remodeling of the cartilage explants. PMA, phorbol 12-myristate-13-acetate.

The overall experimental procedure is presented in Fig. 1.

### 2.1 Isolation of human CD4^+^ T cells

Buffy coats were used from anonymous Danish healthy blood donors obtained at Copenhagen University Hospital. Peripheral blood mononuclear cells (PBMCs) were isolated from buffy coats by Ficoll-plague (Sigma-Aldrich, St. Louis, USA) density gradient centrifugation according to the manufacturer's protocol. Then, PBMCs were collected, and any remaining red blood cells were lysed by ACK lysing buffer (Gibco, Waltham, USA). CD4^+^ T cells were isolated from the PBMCs by negative magnetic selection using human CD4^+^ T cell isolation kit according to the manufacturer's protocol (Miltenyi Biotec, Bergisch Gladbach, Germany).

### *2.2* *In vitro* CD4^+^ T cell differentiation and activation

The naïve CD4^+^ T cells were cultured in flat bottom 24-well Costar® plates (Corning®, New York, USA) at a cell density of 5 × 10^5^ per well and stimulated with plate-bound anti-human CD3 (1.5 μg/mL, Clone UCHT1, BD Pharmingen, New Jersey, USA) and anti-human CD28 (1 μg/mL, Clone CD28.2, BD Pharmingen, New Jersey, USA). Additionally, cells were stimulated by the Th17 differentiation cocktail consisting of human IL-6 (25 ng/mL, RnD systems, Minneapolis, USA), IL-23 (15 ng/mL, RnD systems, Minneapolis, USA), IL-21 (15 ng/mL, RnD systems, Minneapolis, USA), IL-1β (15 ng/mL, RnD systems, Minneapolis, USA), TGF-1β (1 ng/mL, RnD systems, Minneapolis, USA) with neutralizing monoclonal human anti-IL-4 (12 μg/mL, Clone MP4-25D2, BD Pharmingen, New Jersey, USA), anti-INFγ (12 μg/mL, Clone B27, BD Pharmingen, New Jersey, USA), anti-IL-2 (5 μg/mL, Clone 5334, RnD systems, Minneapolis, USA) in IMDM GlutaMAX™ Supplement medium (Gibco, Waltham, USA) supplemented with 10 % fetal bovine serum (FBS), 2-Mercaptoethanol (50 μM), 1 % Penicillin-Streptomycin. Cells cultured with medium alone served as control. Cells were incubated at humidified 37 °C in 5 % CO_2_ for 5 days. On day 3, fresh culture medium with IL-23 (8 ng/mL) was added to wells, except from the control wells only receiving culture medium.

After 5 days of incubation, cells were washed in culture medium without FBS and transferred to new flat bottom 24-well plates at a cell density of 1 × 10^6^per well. For the Th17 cell differentiation verified using flow cytometry, cells were activated with the cytokine production cocktail containing Phorbol 12-myristate 13-acetate, PMA (50 ng/mL, Sigma-Aldrich, St. Louis, USA), Ionomycin (1 μg/mL, Sigma-Aldrich, St. Louis, USA) and Brefeldin A (1 μL/mL, Sigma-Aldrich, St. Louis, USA) for 4 h at humidified 37 °C in 5 % CO_2_. Inactivated cells were included as control. For the Th17-CM harvest and cytokine quantification by ELISAs, cells were only activated with PMA and Ionomycin to ensure that the produced cytokines were released into the medium. After 4 h of activation, cells analyzed by the flow cytometry were washed and transferred to tubes for fixation and staining while media from the Th17-CM cells were harvested and stored in aliquots at −80 °C prior to ELISA measurements and *ex vivo* experiments. Steps are explained thoroughly in the following sections.

### 2.3 Verification of Th17 differentiation

#### 2.3.1 Flow cytometry

The Th17 differentiated cells, including two controls (Untreated naïve CD4^+^ cells and Th17 cells not activated with PMA, Ionomycin, Bredeldin A) were stained in the presence of a fixable cell viability marker Dye eFluor 450 (eBioscience, San Diego, USA) and the surface marker CD4^+^ by anti-human PE-Cyanine7-conjugated mAb (Clone RPA-T4, eBioscience, San Diego, USA). Next, cells were fixed and permeabilized (BD Cytofix/Cytoperm, BD Biosciences, New Jersey, USA) for intercellular staining of IL-17A, APC-conjugated mAb (Clone 41809, RnD systems, Minneapolis, USA). The surface markers and intracellular cytokines expression of the cell suspensions were studied using a 10-color flow cytometry (Beckman Coulter, Brea, USA), and data were analyzed by Kaluza Analysis Software (Beckman Coulter, Brea, USA). Unstained cells, including single staining and compensation controls were applied to compensate for levels of spectral overlap between the different fluorophores on the multicolor flow cytometry. A minimum of 50,000 events were acquired on the flow cytometry. Th17 cells were defined and analyzed by gating on live, CD4^+^, and IL-17A+ cells.

#### 2.3.2 Quantification of Th17-related cytokines by ELISA

Secreted cytokines from the Th17 cells were quantified in the CM using commercial ELISA kits according to the manufacturer's instructions, human IL-17A (Cat. no. BMS2017, Thermo Fisher Scientific, Inc.), human IL-17F (Cat. no. BMS2037-2, Thermo Fisher Scientific, Inc.), human IL-17A/F (Cat. no. BMS2082, Thermo Fisher Scientific, Inc.), human IL-22 (Cat. no. BMS2047, Thermo Fisher Scientific, Inc.), and human TNFα (Cat. no. BMS223-4, Thermo Fisher Scientific, Inc.). All samples were analyzed as duplicates.

### 2.4 Cartilage explants cultured with Th17-CM and inhibitors

#### 2.4.1 Explant isolation and experimental setup

For each study, bovine cartilage explants were isolated from 1 to 2 year old cows, previously described by Engstrøm et al. (2022) [20]. Immediately after isolation, explants were washed and incubated overnight at 37 °C with 5 % CO2 in culture medium composed of Dulbecco's Modified Eagle Medium (DMEM)/F12-GlutaMAX™ (Invitrogen, MA, USA) with 1 % Penicillin Streptomycin (Sigma-Aldrich, MA, USA) and 2.5 μg/mL Fungizone (Amphotericin B, Sigma-Aldrich). Two studies were established to examine the effect of Th17-CM on cartilage ECM remodeling and further explore the consequences of adding anti-IL-17A and -TNFα treatment to the Th17-conditioned cartilage.

In the first study, explants were randomly divided into six treatment groups consisting of six replicates. Explants were cultured for three weeks in 24-well plates in either 1) culture medium alone (untreated) or 2) with IL-17A (RnD systems, Minneapolis, USA) or in a combination of 3) IL-17A + anti-IL-17A (RnD systems, Minneapolis, USA), or 4) IL-17A + TNFα (Sigma-Aldrich, MA, USA). Furthermore, explant group 5) was treated with the Th17-CM diluted in culture medium (1:2) and group 6) received Th17-CM (1:2) with anti-IL-17A treatment (Table 1). In the second study, explants were randomly divided into seven treatment groups consisting of eight replicates and cultured for three weeks in 24-well plates. Group 1) received culture medium alone (untreated), 2) IL-17A, 3) TNFα, 4) TNFα + anti-TNFα (RnD systems, Minneapolis, USA), and 5) a combination of IL-17A + TNFα. Furthermore, group 6) received the Th17-CM diluted in culture medium (1:2) and group 7) Th17-CM (1:2) with anti-TNFα (Table 2). All reagents added to the bovine cartilage explants were specific for human. The IL-17A concentration applied in each study was added to match the IL-17A concentration measured by ELISA in the Th17-CM of batch#1 and #2, respectively. Hence, the same IL-17A concentration of Th17-CM diluted 1:2 was added to the control groups. The concentration of anti-IL-17A was chosen based on suppliers' recommendations. In the first experiment, TNFα concentration was chosen based on previous studies where bovine cartilage explants treated with TNFα and Oncostatin M (OSM) showed to induced catabolic response [[21], [22], [23]]. In the second experiment, the TNFα concentration was added to match the TNFα concentration measured in the Th17-CM of batch#2 diluted 1:2. The concentration of anti-TNFα was chosen based on suppliers’ recommendations. For each study, the supernatant was collected three times a week, and fresh culture medium and treatments were added to the explants. The metabolic activity of the cells was assessed by the Alamar Blue assay, explained in section 2.4.2. The collected supernatant was frozen at −80 °C and stored for use in tissue biomarker analysis, explained in section 2.4.3.

**Table 1:** **Treatment groups of study 1.** *Explant concentration per plate-well. CM, conditioned media.

Study 1		
Treatment no.	Treatment	Concentration*
1	Untreated (Culture medium)	–
2	IL-17A	[1.4 ng/mL]
3	IL-17A + anti-IL-17A	[1.4 ng/mL] + [0.1 μg/mL]
4	IL-17A + TNFα	[1.4 ng/mL] + [20 ng/mL]
5	Th17-CM	CM diluted 1:2 with culture medium, corresponding to IL-17A [1.4 ng/mL]
6	Th17-CM + anti-IL-17A	CM diluted 1:2 with culture medium, corresponding to IL-17A [1.4 ng/mL] + anti-IL-17A [0.10 μg/mL]

**Table 2:** **Treatment groups of study 2.** *Explant concentration per plate-well. CM, conditioned media.

Study 2		
Treatment no.	Treatment	Concentration*
1	Untreated (Culture medium)	–
2	IL-17A	[1.1 ng/mL]
3	TNFα	[2.9 ng/mL]
4	TNFα + anti-TNFα	[2.9 ng/mL] + [1 μg/mL]
5	IL-17A + TNFα	[1.1 ng/mL] + [2.9 ng/mL]
6	Th17-CM	CM diluted 1:2 with culture medium, corresponding to IL-17A [1.1 ng/mL], TNFα [2.9 ng/mL]
7	Th17-CM + anti-TNFα	CM diluted 1:2 with culture medium, corresponding to IL-17A [1.1 ng/mL], TNFα [2.9 ng/mL], + anti-TNFα [1 μg/mL]

#### 2.4.2 Alamar Blue

The metabolic activity of the cells within each explant was measured during the first experiment day and then once weekly by the Alamar Blue assay (Invitrogen, MA, USA). Explants were incubated with 10 % Alamar Blue mixed in culture medium for 3 h, 37 °C, 5 % CO2. Active metabolic cells reduce the Alamar Blue medium from a weakly fluorescent blue state into a fluorescent pink state [24]. For each explant, the reduced Alamar Blue medium was transferred into a black microtiter plate and fluorescence signals were measured at an excitation wavelength at 540 nm and an emission wavelength at 590 nm. Four control wells with no tissue were likewise measured to adjust for background-fluorescence. Subsequently, explants were washed three times in culture medium before addition of new treatments.

#### 2.4.3 Quantification of tissue biomarkers

To examine cartilage ECM remodeling in response to Th17-CM treatment and IL-17A and TNFα inhibitors, two neo-epitope biomarkers (C2M, AGNx1) were measured in the cartilage supernatant with ELISAs. The competitive ELISA, C2M (nordicC2M^TM^, Nordic Bioscience, Herlev, Denmark) was measured to quantify MMP-mediated type II collagen degradation. C2M targets the MMP-1, -2, -9, -13 generated neo-epitope fragment (KPPGRDGAAG^1053^) released from the C-terminal region of the triple helical domain of type II collagen [25]. Aggrecan degradation was measured with the AGNx1 competitive ELISA (nordicAGNx1^TM^, Nordic Bioscience, Herlev, Denmark) which recognizes the C-terminal peptide (NITEGE^373^) released by the aggrecanases ADAMTS-4 and -5 from the aggrecan G1 domain [26]. The fibronectin remodeling was assessed by the FBN-C direct ELISA (nordicFBN-C^TM^, Nordic Bioscience, Herlev, Denmark) measuring the C-terminal end of fibronectin (VQADREDSRE) [27]. Lastly, type II collagen formation was measured by the PRO-C2 competitive ELISA (NordicPRO-C2^TM^, Nordic Bioscience, Herlev, Denmark), measuring the N-terminal pro-peptide (QDVRQPG) of type IIB procollagen, named PIIBNP, which is a procollagen fragment from the synthesis of type II collagen (Table 3) [28]. The C2M, AGNx1, FBN-C, and PRO-C2 ELISA kits are specific for both human and bovine. For the ELISA assays, the biomarker concentrations were calculated from a standard curve included on each plate measured. Previous studies have suggested that ADAMTSs are more rapidly activated in cartilage tissue compared to MMPs, therefore, the AGNx1 results are presented from day 3, 5, 7, and 14, while C2M, FBN-C, and PRO-C2 are displayed from day 14 and 21 [23,26]. Biomarker measurements from all days can be found in supplementary.

**Table 3:** **Overview of measured biomarkers representingECMremodeling**. ECM, extracellular matrix; MMP, Matrix-metalloproteinase; ADAMTS, A Disintegrin and Metalloproteinase with Thrombospondin motifs.

Biomarker	Target	Measure	Reference
C2M	MMP-1, -2, -9, -13 mediated degradation of type II collagen	Type II collagen degradation	[25]
AGNx1	ADAMTS-4 and -5 mediated degradation fragment of aggrecan	Aggrecan degradation	[26]
FBN-C	C-terminal end of fibronectin	Fibronectin remodeling	[27]
PRO-C2	N-terminal propeptide of type II collagen (PIIBNP)	Type II collagen formation	[28]

### 2.5 Statistical analysis

Statistical analyzes were performed using the GraphPad Prism 10.0.2 software (San Diego, California), including the calculation of mean with standard deviation (SD), median with interquartile range, and *p*-values. In study 1 and 2, the differences in the mean values between treatment groups were compared using a two-way analysis of variance (2-way ANOVA) with repeated measurements for the different timepoints (days of culture). Fisher's Least Significant Difference (LSD) test was applied since each comparison between treatment groups stands alone. Each treatment group in study 1 contained six explant replicates (n = 6) while study 2 consisted of eight replicates (n = 8). Treatment groups were considered significantly different when *p*-value was less than 0.05. Biomarker data from each treatment group is presented as bars with median and interquartile range.

## 3 Results

### 3.1 Verification of Th17 differentiation

**Fig. 2:**
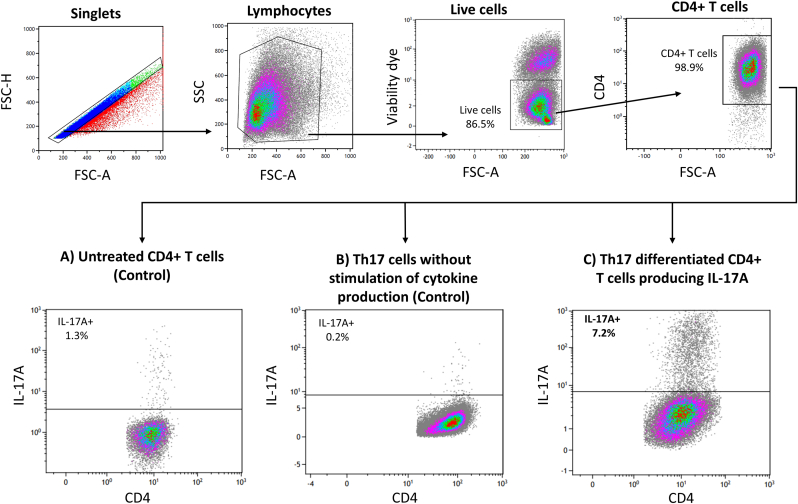
**Identification and verification of IL-17A producing CD4**^**+**^**cells.** Singlets cells were gated according to the forward scatter height (FSC–H) vs. forward scatter area (FSC-A) density plot to exclude doublets. Debris were excluded by gating on lymphocytes according to side scatter (SSC) vs. FSC-A density plot, and further dead cells were excluded by gating on live cells applying viability dye. The CD4^+^ T cells were gated by surface marker CD4^+^ labeling. The production of IL-17A from untreated CD4^+^ cells (control, Fig. 2A), Th17 differentiated CD4^+^ cells not receiving cytokine production cocktail (control, Fig. 2B), and finally Th17 differentiated CD4^+^ cells with cytokine production cocktail to secrete IL-17A (Fig. 2C) was measured by intracellular labelling with IL-17A specific antibodies. The IL-17A producing CD4^+^ cells originate from buffy coat from three healthy human donors with same blood type.

For the control groups, 1.3 % of the untreated CD4^+^ T cells produced IL-17A (Fig. 2A) while 0.2 % of the Th17 cells not receiving cytokine production cocktail secreted IL-17A (Fig. 2B). Finally, 7.2 % of the Th17 differentiated CD4^+^ T cells (receiving cytokine production cocktail) produced IL-17A (Fig. 2C).

### 3.2 Levels of cytokines in the Th17-CM

**Fig. 3:**
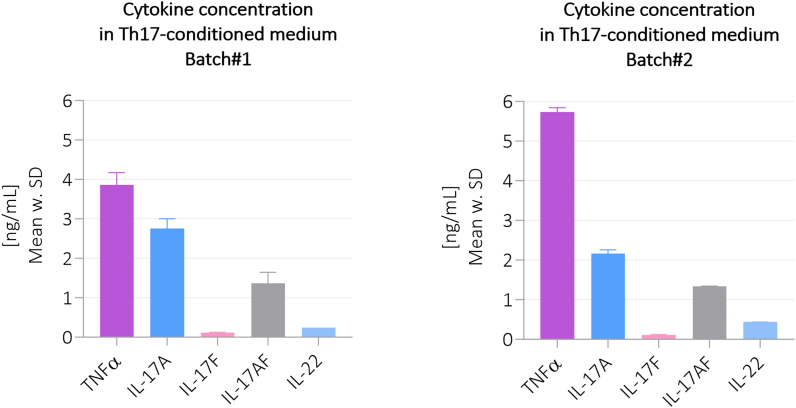
**Cytokine concentrations inthe Th17-CM of batch#1 and batch #2.** The presented data of each cytokine concentration measured in batch#1 and batch#2 consist of two measurements (n = 2). Concentrations are shown as mean w. SD. SD, standard deviation.

Two batches of Th17-CM were produced to ensure an adequate amount of treatment volume for the following *ex vivo* culturing experiments. For each batch, three portions of buffy coat donors were required with individual CD4^+^ T cell isolation, Th17 differentiation and activation, including verification using flow cytometry. The cytokine concentrations of batch#1 and batch#2 showed to be relatively similar, despite different healthy donors were applied (Fig. 3, Table 4).

**Table 4:** **Cytokine concentrations of Th17 conditions medium of batch#1 and batch #2.** The presented data of each cytokine concentration measured in batch#1 and batch#2 consist of two measurements (n = 2). Concentrations are shown as mean w. SD. SD, standard deviation.

	TNFα	IL-17A	IL-17F	IL-17AF	IL-22
Batch#1	3.86 ± 0.31	2.76 ± 0.25	0.12 ± 0.01	1.36 ± 0.28	0.24 ± 0.00
Batch#2	5.74 ± 0.11	2.17 ± 0.09	0.12 ± 0.02	1.34 ± 0.01	0.44 ± 0.01

### 3.3 ECM biomarker profiles in Th17-conditioned cartilage treated with inhibitors

#### 3.3.1 Study 1

**Fig. 4:**
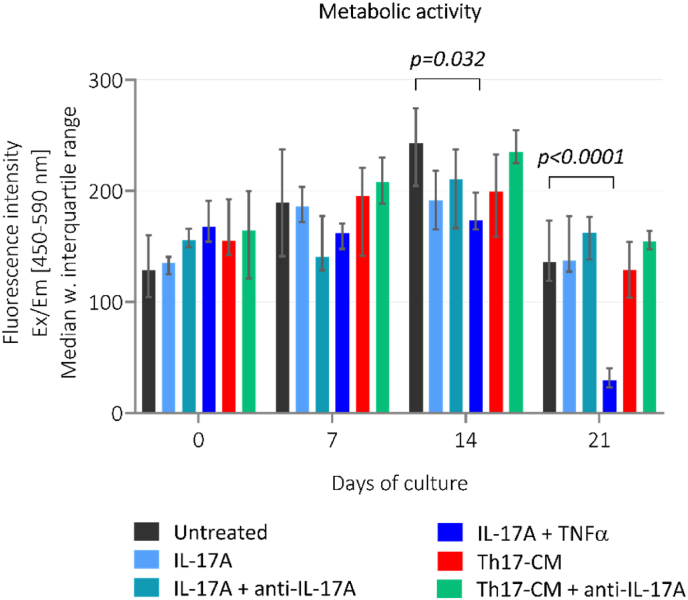
**The metabolic activity assessed in cartilage explants from study 1.** The metabolic activity of cartilage explants was measured in supernatant by Alamar Blue on day 0, 7, 14, and 21. Treatment groups are presented as bars with median and interquartile range, where each group consists of six cartilage explants, n = 6. Th17-CM, Th17-conditioned media. (For interpretation of the references to color in this figure legend, the reader is referred to the Web version of this article.)

From day 14 to 21, the explants treated with IL-17A + TNFα demonstrated a decrease in the metabolic activity compared to untreated group (*p*=0.032, *p*<0.0001). For the remaining groups the metabolic activity of the explants stayed stable throughout the study (Fig. 4).

**Fig. 5:**
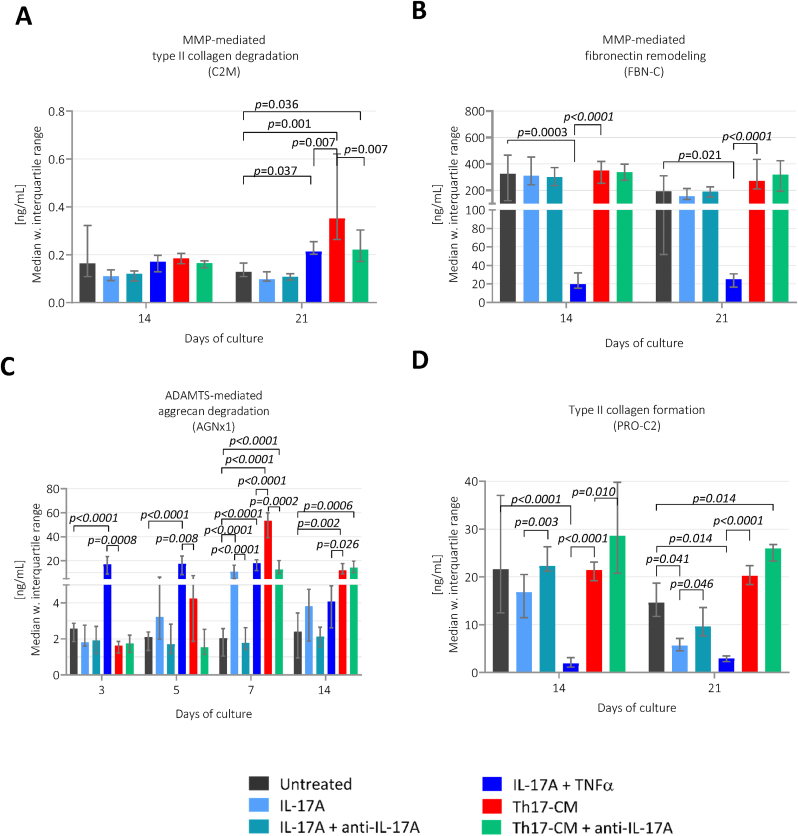
**The cartilage remodeling profile assessed by ECM biomarkers in study 1.** The biomarkers levels of A) C2M, B) FBN-C, D) PRO-C2 measured in the supernatant are presented from day 14 and 21, while measurements of C) AGNx1 are presented from day 3, 5, 7, and 14. Treatment groups are presented as bars with median and interquartile range, where each group consists of six cartilage explants, n = 6. Th17-CM, Th17-conditioned media.

The explants were treated with Th17-CM to explore if Th17-CM stimulated a catabolic or/and anabolic response in the cartilage structure compared to the untreated group. C2M was elevated on day 21 for Th17-CM compared to untreated group (*p*=0.001, Fig. 5A). FBN-C was not significantly elevated in response to Th17-CM compared to untreated group (Fig. 5B). AGNx1 was elevated in Th17-CM treated group compared to untreated group on day 7 (*p*<0.0001) and day 14 (*p*=0.002 , Fig. 5C). The anabolic biomarker, PRO-C2, was not significantly altered in cartilage treated with Th17-CM compared to untreated group (Fig. 5D).

##### 3.3.1.1 Effects of anti-IL-17A treatment in Th17-conditioned cartilage

One group of explants treated with Th17-CM also received repeated doses of anti-IL-17A to investigate its potential to inhibit the catabolic processes activated by Th17-CM in the cartilage. On day 21, the C2M levels were reduced in group receiving Th17-CM + anti-IL-17A treatment compared to group only receiving Th17-CM (*p*=0.007). However, C2M levels measured in Th17-CM + anti-IL-17A treated group were still higher than the untreated group (*p*=0.036, Fig. 5A), indicating that the activated type II collagen degradation has not been reduced to untreated control levels. FBN-C levels were not altered by the anti-IL-17A treatment which agrees with the lack of effect of the Th17-CM on the explants compared to untreated group (Fig. 5B). The Th17-CM + anti-IL-17A treatment reduced the AGNx1 levels compared to Th17-CM treated group on day 7 (*p*=0.0002), although levels were still higher than untreated (*p*<0.0001). On day 14, the anti-catabolic effect by anti-IL-17A diminished (Fig. 5C). Lastly, PRO-C2 was elevated on day 14 in explants receiving anti-IL-17A with Th17-CM compared to Th17-CM alone (*p*=0.010 , Fig. 5D).

#### 3.3.2 Study 2

**Fig. 6:**
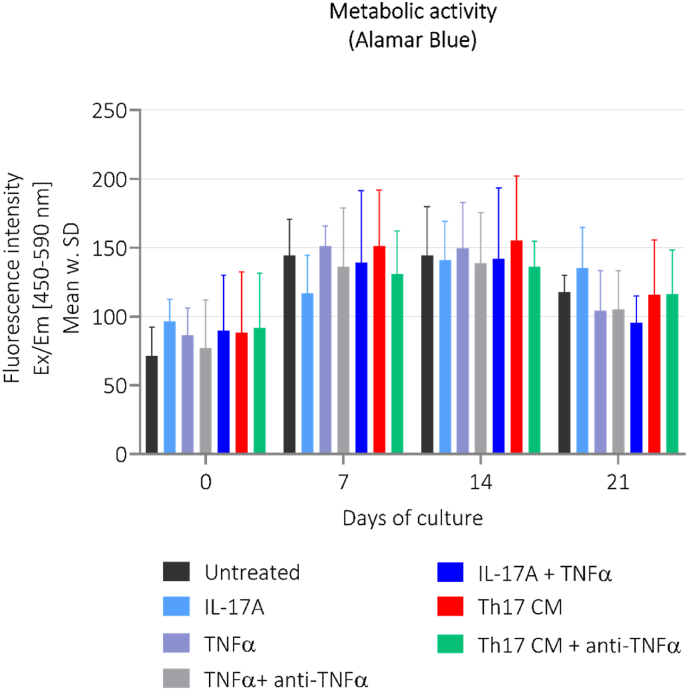
**The metabolic activity assessed in cartilage explants from study 2.** The metabolic activity of cartilage explants was measured in supernatant by Alamar Blue on day 0, 7, 14, and 21. Treatment groups are presented as bars with median and interquartile range, where each group consists of eight cartilage explants, n = 8. Th17-CM, Th17-conditioned media. (For interpretation of the references to color in this figure legend, the reader is referred to the Web version of this article.)

The metabolic activity remained stable throughout the study (Fig. 6).

Study 2 was established to investigate the catabolic effect of the Th17-CM on cartilage explants, particularly regarding TNFα and its potent ability to drive the ECM degradation. With this model we aimed to investigate if IL-17A and TNFα are the main catabolic drivers of the Th17-CM, and further the effect on the interplay between the Th17-derived cytokines when only inhibiting TNFα.

##### 3.3.2.1 Comparison of the stimuli activated by Th17-CM and IL-17A + TNFα

**Fig. 7:**
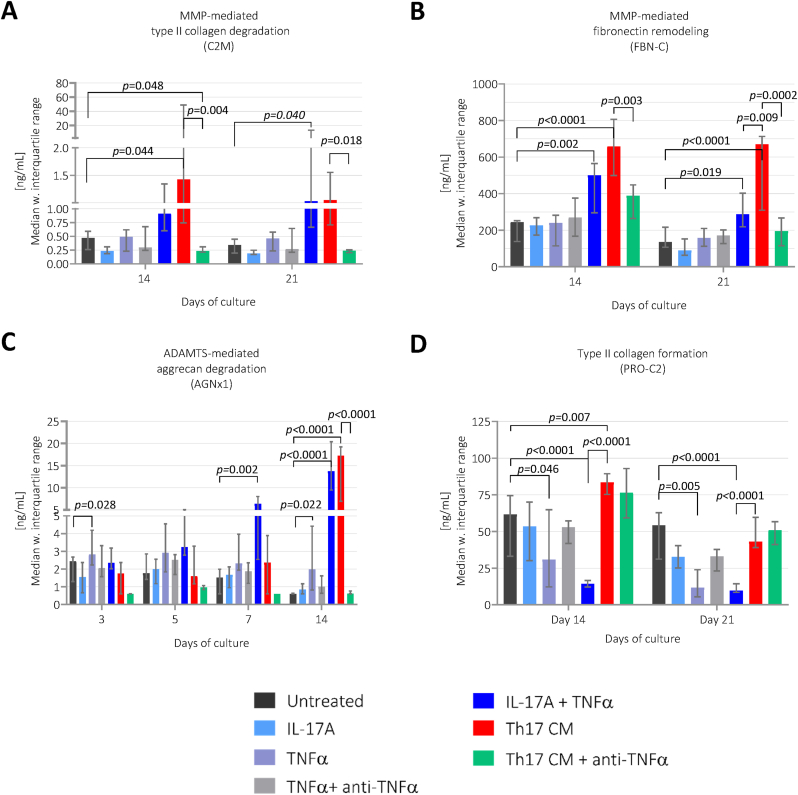
**The cartilage remodeling profile assessed by ECM biomarkers in study 2.** The biomarkers levels of A) C2M, B) FBN-C, D) PRO-C2 measured in the supernatant are presented from day 14 and 21, while measurements of C) AGNx1 are presented from day 3, 5, 7, and 14. Treatment groups are presented as bars with median and interquartile range, where each group consists of eight cartilage explants, n = 8. Th17-CM, Th17-conditioned media.

The Th17-CM did not stimulate significantly higher levels of C2M compared to the stimuli by IL-17A + TNFα (Fig. 7A). On day 21, the FBN-C levels were higher in explants treated with Th17-CM compared to IL-17A + TNFα (*p*=0.009, Fig. 7B). On day 7, the IL-17A + TNFα treatment activated elevated AGNx1 levels compared to untreated group (*p*=0.002). On day 14, the AGNx1 levels in Th17-CM and IL-17A + TNFα were both similarly elevated compared to the untreated group (*p*<0.0001, Fig. 7C). Investigating the anabolic profile, IL-17 + TNFα reduced the type II collagen formation compared to Th17-CM treated group (*p*<0.0001, Fig. 7D), as observed in study 1 (Fig. 5D).

##### 3.3.2.2 Effects of anti-TNFα treatment in Th17-conditioned cartilage

The addition of anti-TNFα to the Th17-CM reduced C2M levels on day 14 (*p*=0.004) and on day 21 (*p*=0.018), to levels even lower than untreated group on day 14 (*p*=0.048, Fig. 7A). The anti-TNFα treatment also reduced FBN-C on day 14 (*p*=0.003, Fig. 7B) and day 21 (*p*=0.0002, Fig. 7B) and AGNx1 on day 14 (*p*<0.0001, Fig. 7C) compared to Th17-CM treated group. PRO-C2 was not significantly affected by the anti-TNFα, which may confirm that TNFα do not play an anabolic role (Fig. 7D).

## 4 Discussion

Therapeutic targeting of IL-17A has shown substantial promise as treatment of psoriasis, PsA, and AS, which has revolutionized the management of these inflammatory diseases, leading to improved clinical outcomes for the patients. However, despite the clinical efficacy observed, our understanding of the effects of anti-IL-17A treatment on the ECM remodeling in the affected tissues remains incomplete. The anti-IL-17A treatment may also have an impact on other inflammatory pathways such as those controlled by other Th17-derived cytokines, such as IL-17F, IL-22, and TNFα, further influencing the ECM dynamics. To better understand how anti-IL-17A treatment affects the ECM synthesis and degradation in a Th17-conditioned inflammatory milieu, we aimed to establish an indirect Th17 cell model where the effects of anti-IL-17A and anti-TNFα treatment on joint tissue remodeling were analyzed with protease-mediated ECM biomarkers.

### 4.1 Differentiation and verification of Th17 cells

Differentiation of naïve CD4^+^ T cells is a very complex process, requiring a definite cytokine milieu determining the effector lineage that the cell will assume, by inducing lineage-specific transcription factors. In this study, naïve CD4^+^ T cells were stimulated in presence of anti-CD3 and -CD28, together with IL-6, IL-23, IL-21, IL-1β, TGF-1β which have proven to be required for Th17 differentiation [29,30]. Furthermore, neutralizing monoclonal antibodies were added (anti-IL-4, anti-INFγ, anti-IL2) to prevent the naïve CD4^+^ T differentiating into Th1, Th2, and T regulatory cells (Tregs). Although our main goal was to successfully generate CD4^+^ T cells which produced IL-17A but also IL-17F and IL-22, confirming Th17 differentiation, previous studies have indicated that the functional heterogeneity of Th17 subsets is much more complex. New subsets of non-pathogenic IL-10-producing Th17 cells have been discovered in mice by McGeachy et al. (2007). Here, stimulation with only IL-6 and TGF-β promoted generation of non-pathogenic Th17 cells producing IL-17 but failed to upregulate the expression of proinflammatory chemokines, instead Th17 cells secreted IL-10. Study also showed that stimulation with only IL-23 resulted in more pathogenic Th17 cells expressing IL-17 and proinflammatory chemokines but not IL-10 [31]. As it has become clear that there are many faces of Th17 cells dependent on the surrounding stimulating milieu, Th17 differentiation has shown to be a long and winding road. Thus, generating inflammatory Th17 cells acting with similar pathogenicity seen in autoimmunity is very challenging and may be due to the lack of methods that recapitulate niche tissue conditions during autoimmunity. We intended to generate inflammatory Th17 cells secreting IL-17A, but it cannot be ruled out that the differentiated Th17 cells consist of different subtypes of Th17 cells, some pathogenic and some non-pathogenic. Hence, explaining the trend of anti-inflammatory appearance in the Th17-CM.

The Th17 differentiation was optimized to achieve the highest yield of Th17 cells producing IL-17A. The verification of Th17 cell differentiation using flow cytometry revealed that 7.2 % of the CD4^+^ T cells produced IL-17A. To our knowledge, literature verifying Th17 cell differentiation from isolated healthy human CD4^+^ T cells using flow cytometry is limited. However, in studies where human, mice, or bovine CD4^+^ T cells underwent comparable Th17 polarization conditions similarly to our study, 2.7 %, 5.5 %, or 3.5 % of CD4^+^ T cells produced IL-17 [[32], [33], [34]]. The 7.2 % in our study is therefore similar to previous studies using this method. One possible explanation of the relative low percentage could be a result of negative feedback loop limiting Th17 pathogenicity as suggested by Chong et al. (2020), where their study showed that IL-17A can bind its own receptor on Th17 cells, activating signaling cascade which leads to IL-24 secretion, repressing the Th17 cytokine program [35].

Following the analysis of Th17 differentiation using flow cytometry, the actual Th17-derived cytokine concentrations were quantified by ELISA in the Th17-CM. Here, TNFα and IL-17A were secreted in highest concentrations by the Th17 cells, followed by IL-17AF, IL-22, and IL-17F in lower amounts, which further confirms the Th17 differentiation by detecting Th17-specific cytokines [36]. Interestingly, TNFα is the most abundant cytokine in both batches of Th17-CM. This may be a result of that naïve T cells upon activation can produce TNFα during maturation state, prior to acquiring other effector functions [37].

Subsequently, the Th17-CM were added to bovine cartilage explant cultures to investigate the ECM remodeling in a Th17-conditioned inflammatory milieu. The quantified cytokine levels in the Th17-CM were used to generate the controls mimicking the most accurate stimuli induced by the Th17-CM. Thus, in batch#1 the IL-17A concentration in Th17-CM was 2.76 ng/mL (∼2.8 ng/mL) and Th17-CM was added in 1:2 dilution, therefore 1.4 ng/mL of IL-17A was added to the IL-17A control group.

### 4.2 Effects of IL-17A and TNFα inhibitor in Th17-conditioned cartilage

In cartilage explants stimulated with Th17-CM, degradation of aggrecan and type II collagen was elevated in both study 1 and study 2, without inducing type II collagen formation. Interestingly, type II collagen formation levels in Th17-CM treated group were similar to the untreated group, while IL-17A and TNFα treatment significantly reduced type II collagen formation. This may suggest that IL-17A and TNFα treatment drives a more catabolic response, while the Th17-CM may contain anabolic or anti-inflammatory factors affecting the Th17-derived cytokine cocktail. While IL-17A and TNFα are widely studied pro-inflammatory cytokine, less is known about the inflammatory effects of IL-17F and IL-22. Although studies in keratinocytes and colonic myofibroblasts have shown that IL-22 activates the release of anti-microbial proteins, acute-phase proteins, inflammatory cytokines, and chemokines [[38], [39], [40]], others have found that IL-22 also exhibits anti-inflammatory roles by protecting hepatocytes from damage during acute liver inflammation [41,42]. IL-22 shares structural homology with the anti-inflammatory cytokine IL-10 and signals through the IL-22 receptor complex consisting of the high-affinity subunit, IL-22Ra, and the low-affinity subunit, IL-10Rβ. It has been suggested, that tissue-selective modulation of STAT1/3 signaling through the IL-22 receptor complex can switch between tissue-protective and pro-inflammatory functions of IL-22 [43]. Thus, IL-22 produced by Th17 cells may enhance inflammation or limit tissue damage induced by the other Th17-derived cytokines such as IL-17A.

In study 1, anti-IL-17A inhibited type II collagen and aggrecan degradation levels only moderately, with levels still increased compared to untreated. Fibronectin remodeling levels were similar between untreated and Th17-CM treated group, and not surprisingly the levels remained unaffected by IL-17A inhibition. In contrast, in study 2 anti-TNFα inhibited type II collagen and aggrecan degradation to the level of untreated explants, as with fibronectin remodeling. These results indicate that IL-17A inhibition reduced degradation markers partly, while TNFα inhibition reduced them completely. This could indicate that IL-17A inflammatory signaling is dependent on TNFα, while TNFα is not necessarily dependent on IL-17A to exert its pro-inflammatory effect.

We aimed to develop an inflammatory Th17-conditioned environment in a cartilage explant model to examine the ECM synthesis and degradation when adding anti-IL-17A and anti-TNFα treatment. Adding Th17-CM to the cartilage explants instead of purchasing the individual cytokines and combine them into a Th17 ‘like’ treatment served several advantages and disadvantages. The advantage of using Th17-CM was that the Th17-CM contained a complex mixture of cytokines and other molecules that was produced by Th17 cells *in vivo* and may better mimic the inflammatory microenvironment in the joint compared to treating with individual cytokines. On the other hand, applying Th17-CM made the model much more complex, time-consuming, and experimental challenging. The composition of Th17-CM could vary depending on factors such as the use of healthy donors, culture conditions, Th17 cell differentiation, and activation. Altogether, these factors could be difficult to control. However, we successfully managed to produce comparable cytokine concentrations measured in both batches of the Th17-CM. In addition, the Th17-CM contained several unknown cytokines and other molecules that we did not know the effect of and thus, it could be difficult with certainty to interpretate the results. However, the presence of the unknown cytokines and molecules more likely makes Th17-conditioned microenvironment more physiologically relevant.

To our knowledge, inhibitors of IL-17A and TNFα have never been tested before in a cartilage explant model reflecting Th17-conditioned microenvironment considering the potential interactions between multiple cytokines. These complex interactions between multiple cytokines did show in this study, that treatment with anti-IL-17A alone does not fully reduce the accelerated type II collagen and aggrecan degradation, hence, anti-IL-17A treatment may need to be combined with other inhibitory treatment such as anti-TNFα to fully stop the cartilage destruction. As the synovial joint consists of several other types of tissue besides cartilage such as the synovial membrane and bone, future experiments should also test Th17-CM on these tissues to fully elucidate the inflammatory microenvironment activated by Th17 cells in the joint as a complete organ where cytokine from the different tissue also make interactions.

## 5 Conclusions

In this study, we aimed to investigate the effect of anti-IL-17A and anti-TNFα treatment on ECM remodeling in a cartilage model with an inflammatory Th17-conditioned microenvironment. We find that inhibition of IL-17A in Th17-conditioned cartilage tissue only moderately reduced MMP-mediated type II collagen degradation and ADAMTS-mediated aggrecan degradation, while anti-TNFα treatment fully blocked MMP- and ADAMTS-mediated ECM degradation. Combining ECM biomarkers with a Th17-conditioned *ex vivo* model may hold great translational potential as output for describing joint disease mechanisms and predicting structural effects of treatment on joint tissue. Additionally, these results may support the further treatment selection strategy targeting IL-17A, TNFα, or other Th17-related cytokines, ultimately improving the management of rheumatic diseases by identifying the biologically rational treatment or combination for the patients.

## Ethics approval and consent to participate

The use of buffy coats from anonymous Danish blood donors, obtained at Copenhagen University Hospital, was covered by the general ethical approval for research use of donor material in agreement with the Transfusion Medicine Standards (TMS) of the Danish Society of Clinical Immunology (DSKI). These individuals are residents of central Copenhagen and provided written consent to have their blood used for research purposes as normal material. All data were analyzed anonymously. Donors participated in compliance with the Helsinki Declaration.

## Funding

Funding was provided by Den Danske Forskningsfond.

## CRediT authorship contribution statement

**Solveig Skovlund Groen:** Data curation, Funding acquisition, Investigation, Methodology, Visualization, Writing – original draft, Writing – review & editing. **Anne-Christine Bay-Jensen:** Supervision, Writing – review & editing. **Christian S. Thudium:** Methodology, Supervision, Writing – review & editing. **Morten H. Dziegiel:** Supervision, Writing – review & editing. **Marie Skougaard:** Methodology, Writing – review & editing. **Simon Francis Thomsen:** Conceptualization, Supervision, Writing – review & editing. **Signe Holm Nielsen:** Data curation, Investigation, Methodology, Project administration, Supervision, Writing – review & editing.

## Declaration of competing interest

The authors declare the following financial interests/personal relationships which may be considered as potential competing interests:Solveig Skovlund Groen reports financial support was provided by Den Danske Forskningsfond. Anne-Christine Bay-Jensen, Signe Holm Nielsen, Christian S. Thudium reports a relationship with Nordic Bioscience that includes: employment and equity or stocks. If there are other authors, they declare that they have no known competing financial interests or personal relationships that could have appeared to influence the work reported in this paper.

## Data Availability

No data was used for the research described in the article.
