# Supplementary material for: Evaluating the inhibition of IL-17A and TNFα in a cartilage explant model cultured with Th17-derived cytokines
Source: J Transl Autoimmun. 2024 Jan 7;8:100231. doi: 10.1016/j.jtauto.2024.100231 (PMC10826309; doi:10.1016/j.jtauto.2024.100231)
Supplement: Multimedia component 1 [file mmc1.docx]

Supplementary results

Study 1

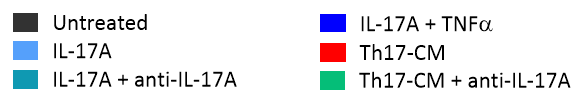


**Figure 1.** **The** **Cartilage remodeling profile assessed by ECM biomarkers in study 1**. The biomarkers levels of A) C2M, B) FBN-C, C) AGNx1, D) PRO-C2 measured in the supernatant from study 1. Treatment groups are presented as bars with median and interquartile range, where each group consists of six cartilage explants, n=6. Th17 CM, Th17 conditioned media.

Study 2

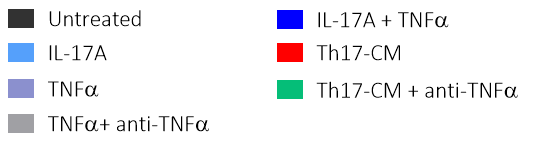


**Figure 2. The** **Cartilage remodeling profile assessed by ECM biomarkers in study 2**. The biomarkers levels of A) C2M, B) FBN-C, C) AGNx1, D) PRO-C2 measured in the supernatant from study 2. Treatment groups are presented as bars with median and interquartile range, where each group consists of eight cartilage explants, n=8. Th17 CM, Th17 conditioned media.
